# A Zebrafish Model of Roberts Syndrome Reveals That Esco2 Depletion Interferes with Development by Disrupting the Cell Cycle

**DOI:** 10.1371/journal.pone.0020051

**Published:** 2011-05-26

**Authors:** Maren Mönnich, Zoë Kuriger, Cristin G. Print, Julia A. Horsfield

**Affiliations:** 1 Department of Pathology, Dunedin School of Medicine, The University of Otago, Dunedin, New Zealand; 2 Department of Molecular Medicine and Pathology, School of Medical Sciences, and the Bioinformatics Institute, The University of Auckland, Auckland, New Zealand; Indiana University, United States of America

## Abstract

The human developmental diseases Cornelia de Lange Syndrome (CdLS) and Roberts Syndrome (RBS) are both caused by mutations in proteins responsible for sister chromatid cohesion. Cohesion is mediated by a multi-subunit complex called cohesin, which is loaded onto chromosomes by NIPBL. Once on chromosomes, cohesin binding is stabilized in S phase upon acetylation by ESCO2. CdLS is caused by heterozygous mutations in NIPBL or cohesin subunits SMC1A and SMC3, and RBS is caused by homozygous mutations in ESCO2. The genetic cause of both CdLS and RBS reside within the chromosome cohesion apparatus, and therefore they are collectively known as “cohesinopathies”. However, the two syndromes have distinct phenotypes, with differences not explained by their shared ontology. In this study, we have used the zebrafish model to distinguish between developmental pathways downstream of cohesin itself, or its acetylase ESCO2. Esco2 depleted zebrafish embryos exhibit features that resemble RBS, including mitotic defects, craniofacial abnormalities and limb truncations. A microarray analysis of Esco2-depleted embryos revealed that different subsets of genes are regulated downstream of Esco2 when compared with cohesin subunit Rad21. Genes downstream of Rad21 showed significant enrichment for transcriptional regulators, while Esco2-regulated genes were more likely to be involved the cell cycle or apoptosis. RNA *in situ* hybridization showed that *runx1*, which is spatiotemporally regulated by cohesin, is expressed normally in Esco2-depleted embryos. Furthermore, *myca*, which is downregulated in *rad21* mutants, is upregulated in Esco2-depleted embryos. High levels of cell death contributed to the morphology of Esco2-depleted embryos without affecting specific developmental pathways. We propose that cell proliferation defects and apoptosis could be the primary cause of the features of RBS. Our results show that mutations in different elements of the cohesion apparatus have distinct developmental outcomes, and provide insight into why CdLS and RBS are distinct diseases.

## Introduction

The cohesin complex is best known for its crucial function in mediating sister chromatid cohesion during the cell division cycle. Cohesin comprises four core subunits: structural maintenance of chromosomes (SMC) subunits Smc1 and Smc3, plus two non-SMC subunits, Mcd1/Scc1/Rad21, and Scc3/Stromalin (SA). The leading theory for establishment of cohesion is that cohesin forms a large molecular ring that entraps sister DNA strands [1].

The loading of cohesin onto chomosomes and the subsequent establishment of cohesion are separate events. Loading of cohesin onto chromosomes takes place in telophase in most organisms, and is facilitated by a protein complex containing Scc2 (*Nipped-B* in *Drosophila* and *NIPBL* in human) and Scc4/MAU-2 [2], [3], [4]. Once loaded, cohesin exhibits marked differences in residence time on chromosomes, indicating that not all cohesin is stably bound [5], [6]. It is thought that the more stably bound fraction of cohesin has functions in addition to chromosome cohesion, including regulating gene expression [5].

During S phase, stably bound cohesin is converted to a ‘cohesive’ form by interaction with the DNA replication machinery [7], [8], [9], in association with an acetyltransferase known as Ctf7/Eco1 in yeast, and Esco2 in vertebrates [9], [10], [11]. Esco2 acetylates cohesin subunit Smc3 to generate the cohesive form of cohesin, essential for holding sister chromatids together through G2 until M phase [12], [13], [14]. This cohesive form of cohesin has a long-term residency on chromosomes commensurate with the stably bound cohesin fraction [5]. Esco2 also appears to antagonize the activity of a cohesion disestablishment complex containing Pds5 and Wapl [15], [16]. Thus, Esco2 is an important factor in ensuring cohesion persists between sister chromatids until their separation at anaphase.

At anaphase, the Rad21 subunit of cohesin is cleaved by the protease separase, and the cohesin ring is opened, allowing chromosomes to separate [17]. The Smc3 subunit of cohesin can be recycled onto chromosomes at the next cell cycle, but deacetylation of Smc3 by class I histone deacetylase Hos1 is required before this can happen [18], [19], [20]. Thus, deacetylation by Hos1 opposes Esco2′s acetylation activity.

Cohesin has a further important role in DNA double strand break repair. For double strand breaks to be effectively repaired, the cohesive form of cohesin must be established at the location of the break [21]. Stabilization of cohesin at double strand breaks depends on acetylation of the Rad21 subunit by Esco2, plus antagonism of the disassociation complex containing Wapl [22].

Since 2004, it has emerged that human developmental syndromes arise from mutations in proteins responsible for sister chromatid cohesion. Inactivating mutations in ESCO2 cause Roberts Syndrome/SC Phocomelia (RBS/SC) [23], [24], [25], [26]. RBS is characterized by severe growth deficiency, microcephaly, craniofacial abnormalities and mental retardation. SC phocomelia is much milder with less marked limb reduction and survival to adulthood. Both disorders arise from ESCO2 mutations with no apparent genotype/phenotype correlation [25], [26]. Since the exact same ESCO2 mutation can cause Roberts or SC Phocomelia, it has been proposed that both be termed Roberts Syndrome, which would then cover the entire spectrum of pathology [26]. Chromosomes from RBS/SC patients are characterized by precocious sister chromatid separation, particularly at heterochromatic regions of the chromosomes [23], [25]. Cornelia de Lange Syndrome (CdLS), on the other hand, is caused by haploinsufficiency for *NIPBL* or by missense mutations in the *SMC1A* or *SMC3* cohesin subunits [27]. CdLS patients display diverse and highly variable mental deficits and structural abnormalities [28], [29], [30], [31]. In contrast to RBS/SC, chromosome cohesion defects are not associated with CdLS [32]. Despite their shared ontogeny within the sister chromatid cohesion apparatus, RBS/SC and CdLS are distinct syndromes. Nevertheless, they have been grouped into a category of human developmental syndromes known as ‘cohesinopathies’.

There is strong evidence that CdLS is caused by altered expression of developmental genes, rather than by cell cycle anomalies (reviewed in [32], [33], [34]). In *Drosophila*, mouse and zebrafish models analyzing Nipbl or cohesin depletion, specific gene expression changes were observed that were not associated with cell cycle or chromosome cohesion defects [34], [35], [36], [37]. Transcript profiling of lymphoblastoid cell lines from CdLS patients also identified consistent gene expression alterations [38]. Genome-scale mapping of cohesin binding sites provides further evidence that it directly regulates transcription. In *Drosophila*, Nipped-B and cohesin co-localize genome-wide, and associate preferentially with active genes [39], [40]. Furthermore, it was recently demonstrated that cohesin directly regulates the expression of a number of genes in *Drosophila* salivary glands in which endoreduplicating chromosomes do not separate, showing definitively that cohesin regulation of gene expression is entirely separable from its role in the cell cycle [41].

In vertebrates, cohesin appears to regulate gene expression in combination with specific transcriptional regulators, such as CTCF [42], [43], [44], [45], mediator complex [46] and estrogen receptor alpha [47]. The favoured model is that gene regulation by cohesin occurs through its mediation of long-range enhancer-promoter communication [46], [48]. It is thought to be the stable fraction of cohesin that functions in regulating gene expression [5]. It is not known if the acetyltransferase activity of Esco2 contributes to stabilizing cohesin for this function.

Our previous research indicated specific roles for cohesin in regulating gene expression during zebrafish development. We found that cohesin is expressed in both proliferating and non-proliferating cells in the zebrafish embryo [49] and is required for early tissue-specific transcription of *runx1* and *runx3* during embryogenesis [36]. Furthermore, cohesin is required for transcription of *myca*, the zebrafish *c-Myc* ortholog [37].

Cohesin's role in developmental gene expression is thought to contribute to the pathology of CdLS [27], [50]. It is not known how ESCO2 activity influences gene expression to contribute to RBS. In this study, we have again used the zebrafish model, to determine if Esco2 contributes to the same developmental pathways that operate downstream of cohesin activity. Esco2-depleted zebrafish embryos recapitulated many of the phenotypes observed in RBS, including limb (fin) truncations and craniofacial abnormalities. However, we found that, in contrast to loss of Rad21 [37], Esco2 depletion does not result in a strong signature of developmental gene misregulation. Of the few genes regulated in common in response to Esco2 and Rad21 depletion, most were involved in the cell cycle and apoptosis. Furthermore, *myca*, *runx1* and *runx3*, which are all tissue-specifically regulated by cohesin, were not regulated in the same direction in Esco2-depleted embryos; in fact, *myca* was upregulated. Esco2 embryos exhibited a cell cycle block in G2 and strong upregulation of caspase activity, regardless of p53 function.

We suggest that the phenotype caused by Esco2 depletion is primarily due to a paucity of cells resulting from cell death following G2 arrest, and not by the regulation of developmental genes. Our research indicates that although the cohesinopathies arise from mutations in a related family of proteins, individual syndromes within the cohesinopathies can have distinct molecular etiology.

## Results

### Identification and expression pattern of zebrafish *esco2* in zebrafish embryo development

Sequence information on the zebrafish *esco2* gene, on chromosome 20, was available in Ensembl (ENDSARG00000014685) and NCBI (Gene ID: 445395). The zebrafish *esco2* transcript is 2350 base pairs long and encodes 609 amino acids. Its identity was confirmed by comparison with other Esco2 sequences (Fig. S1), which revealed that zebrafish Esco2 is 41% identical to the human and mouse proteins (NCBI BLAST). Phylogenetic analysis grouped zebrafish Esco2 together with Esco2 proteins from other species, closest to the Atlantic salmon (*Salmo salar*) with *Xenopus laevis* as the next closest relative (Fig. S2A). A chromosomal location comparison with mouse and human (Fig. S2B) shows conserved gene synteny of *esco2* between these species.

**Figure 1:**
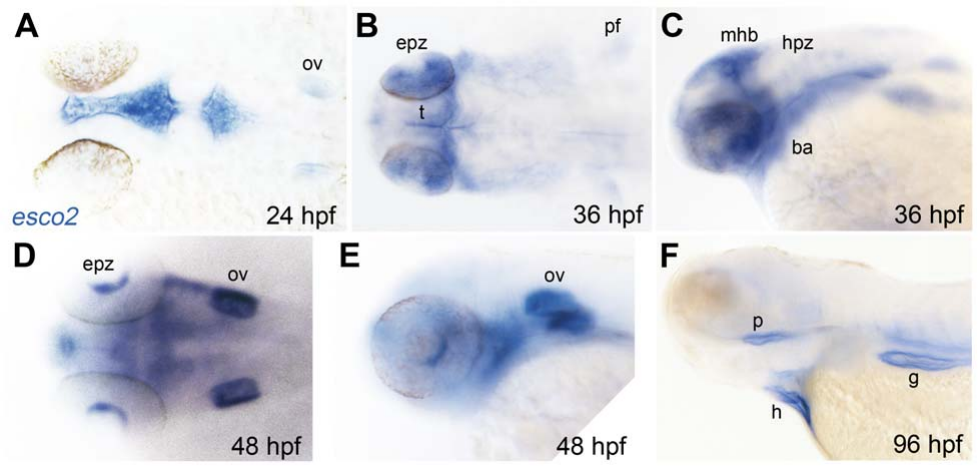
Late embryonic and larval expression of *esco2* in zebrafish. Whole-mount *in situ* hybridization of zebrafish at the indicated stage with an *esco2* antisense riboprobe. A, B, and D are dorsal views, C, E, and F lateral views. Anterior is to the left for all. ov, otic vesicle; epz, eye proliferative zone; pf, pectoral fin; t, tectum; mhb, mid-hindbrain boundary; hpz, hindbrain proliferative zone; ba, branchial arches; p, pharynx; h, heart; g, gut.

Considering their related functions, we first asked if zebrafish *esco2* is expressed in the same cells as cohesin during embryo development. To determine spatiotemporal expression of the *esco2* gene, a riboprobe of the *esco2* gene was hybridized with zebrafish embryos collected at intervals up to 4 days post-fertilization (dpf). *esco2* expression was ubiquitous in early development (data not shown), however from about 24 hours post-fertilization (hpf), specific patterns of *esco2* transcript distribution were observed. From 24–48 hpf, *esco2* was expressed in the brain ventricles and otic vesicles (Fig. 1A–E). From 36–48 hpf, *esco2* is expressed faintly in the developing pectoral fin (Fig. 1B, C) and in a layer of retinal cells (Fig. 1B–E). At 36 hpf, expression of *esco2* was detected in the mid-hindbrain boundary and hindbrain proliferative zone, and in the branchial arches (Fig. 1B, C). At 4 dpf, robust *esco2* expression was detected in the developing pharynx, the gut and the heart (Fig. 1F). In summary, the expression pattern of *esco2* during zebrafish embryo development closely resembles that of cohesin subunits [49], and is predominantly found in proliferative cells. This is consistent with the finding that Esco2 is important for the establishment of sister chromatid cohesion in conjunction with cohesin.

### Esco2-depleted zebrafish exhibit craniofacial defects and reduced pectoral fin growth

Zebrafish embryos were used to evaluate the consequences of Esco2 depletion on early development. We used antisense morpholino oligonucleotides (MOs) targeting the ATG start codon (*esco2*_ATG_MO), or the 5′ donor of the exon/intron boundary of intron 2 of *esco2* (*esco2*_Splx2_MO), to create knockdown “morphant” embryos. RT-PCR amplification of cDNA demonstrated marked reduction in splicing of intron 2 from *esco2* mRNA in *esco2*_Splx2_MO-injected embryos (Fig. S2C), generating an aberrant transcript predicted to lead to early termination of the Esco2 protein. Both MOs elicited identical phenotypes in zebrafish embryos. However, a higher dose of *esco2*_Splx2_MO was needed to generate equivalent effects to *esco2*_ATG_MO (data not shown), perhaps reflecting that the latter MO targets both maternally deposited spliced mRNA as well as zygotically transcribed mRNA. For this reason, *esco2*_ATG_MO (henceforth termed *esco2* MO for simplicity) was used for all subsequent experiments.

**Figure 2:**
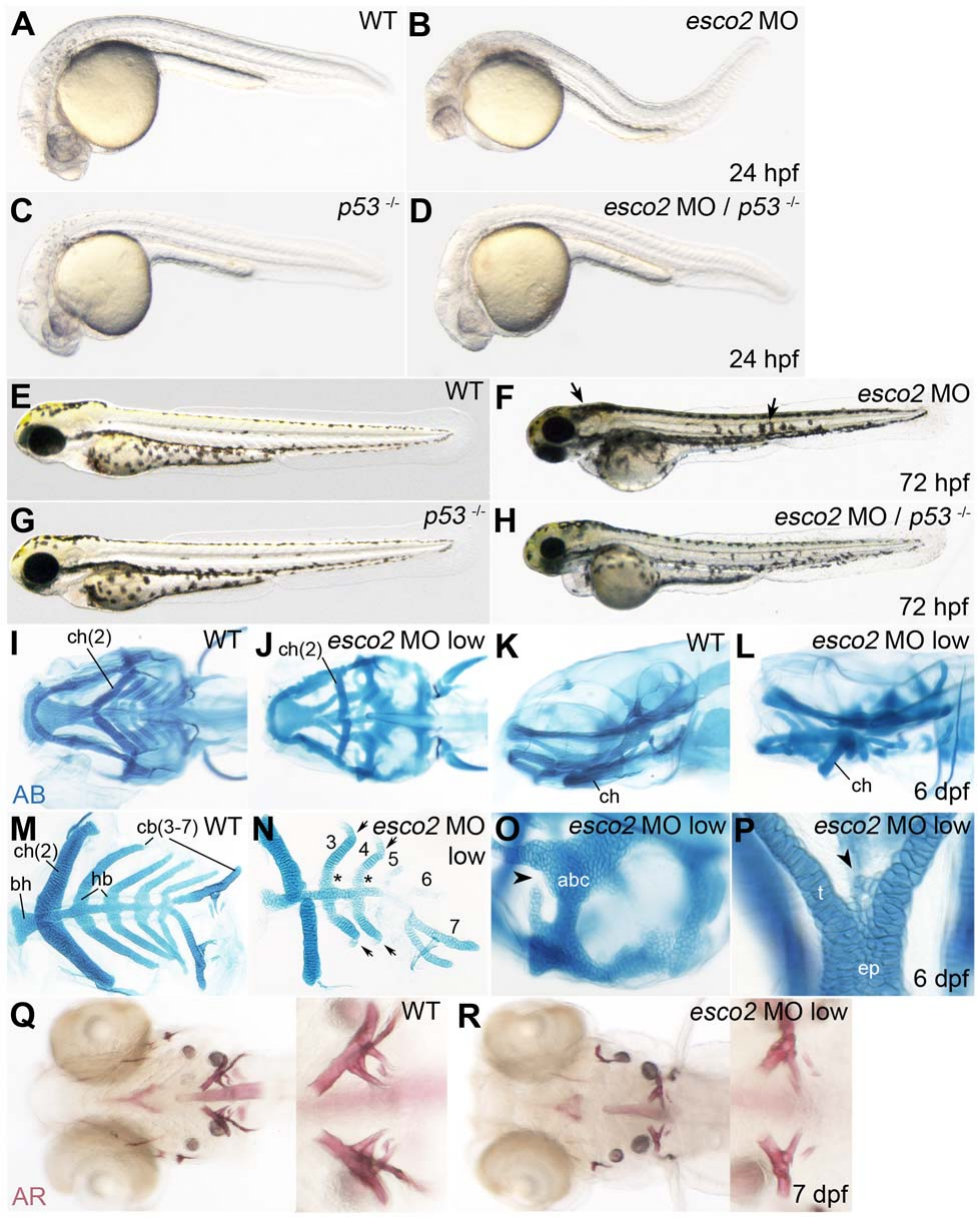
Craniofacial development is abnormal in *esco2* morphants. **A–D**, At 24 hpf, *esco2* morphants (B) had a smaller head, smaller eyes and noticeable cell death in the head region compared to wild type (A), and could be partially rescued by depletion of *p53* (*p53* ^−/−^, C, D). **E–H**, At 72 hpf *esco2* morphants lacked a jaw and showed an abnormal pigmentation pattern (arrows) (F compared with E, wild type). The phenotype was enhanced by depletion of *p53* (G, H). A–H, lateral views of live larvae, anterior to the left. **I–P**, Craniofacial cartilage was stained with Alcian Blue (AB) at 6 dpf. A low dose of *esco2* MO was injected to allow for cartilage development (*esco2* MO low). Numbers of the pharyngeal arch elements are indicated (2–7). I, J, Ventral views. K, L, lateral views. Pharyngeal arch elements were malformed or missing in *esco2* morphants (J, L) compared to wild type (I, K). M, N, closer views of dissected pharyngeal arch elements in wild type (M) and *esco2* morphant (N). The arrows point to the kinks in arches 3 and 4. The asterisks mark elongated hypobranchials (hb) in an *esco2* morphant. O, closer view of anterior basicranial commissure (abc) in an *esco2* morphant, where a gap in the structure was observed (arrowhead). P, closer view of the neurocranium with additional cell structures (arrowhead) at the base of the trabeculae (t) in an *esco2* morphant. **Q**, **R**, Alizarin Red (AR) staining of bone matrix at 7 dpf (ventral views) revealed that ossification is normal in an *esco2* morphant (R, compare to wild type in Q). The close-ups show branchial arch 7. ch, ceratohyal; bh, basihyal; hb, hypobranchial; cb, ceratobranchial; abc, anterior basicranial commissure; t, trabeculae; ep, ethmoid plate.

When compared with wild type controls, *esco2* morphants had smaller eyes and a smaller head at 24 hpf, accompanied by noticeable cell death in the brain (Fig. 2A, B). Somites formed normally but were incorrectly shaped, and there was dorsal curvature of the tail with a thin yolk sac extension. Reduced p53 function modestly rescued morphological abnormalities in 24 hpf *esco2* morphants (Fig. 2C, D). At 72 hpf, *esco2* morphants were slightly smaller than wild type embryos. They had a smaller head that essentially lacked a jaw, showed an abnormal pigmentation pattern, and had reduced blood with circulation defects (Fig. 2E, F). Cardiac and cerebral edema became pronounced at this stage. None of the observed phenotypes was rescued by reduction in p53 function (Fig. 2D, H); in fact, reduction of functional p53 enhanced the *esco2* morphant phenotype (Figs. 2H and S2). *esco2* morphant embryos failed to develop beyond 72 hpf, were highly edemic, and rarely survived beyond this time point.

To gain insight into the craniofacial phenotypes observed in RBS, we wished to observe the effects of *esco2* depletion on cartilage and bone development. To do this, it was necessary to reduce the MO dose to produce hypomorphic *esco2* morphants to allow for cartilage development. *esco2* “hypomorphants” can survive past 72 hpf without the formation of cardiac edema, which might in itself contribute to cartilage malformation. These more mildly affected embryos started to recover after 3 days of development due to the dilution of the morpholino during development, and survived to at least 7 days. *esco2* hypomorphants displayed disorganized craniofacial cartilage with underdeveloped jaw elements, as revealed by Alcian Blue staining. In *esco2* hypomorphants, the ceratohyal arch was oriented ventrally rather than forwards, and ceratobranchial arches 3–6 were reduced in size compared with wild type (Fig. 2I, J, K, L). Ceratobranchial arches 5 and 6 were severely reduced in length and were barely visible with Alcian Blue staining (Fig. 2M, N). Furthermore, the more distal arches were kinked towards anterior at their extremities. The basal element of arch 2 (basihyal of hyoid) was present, but shaped differently in *esco2* hypomorphants than wild type embryos. Hypobranchials were thinner and elongated in *esco2* hypomorphants. In the affected craniofacial cartilage elements, the cell numbers were reduced and cell size was enlarged. In some embryos, gaps in the anterior basicranial commissure could be detected (Fig. 2O), as well as additional cell structures protruding from a cartilage element (Fig. 2P).

Alizarin Red was used to stain for bone formation in *esco2* hypomorphants at 7 dpf. Ossification appeared to be essentially normal in Esco2-depleted embryos (Fig. 2Q, R). The shape of ossified structures was altered relative to wild type, presumably because of the misdevelopment of cartilage in *esco2* hypomorphants.

These data suggest that craniofacial malformations caused by loss of Esco2 could be due to loss and mispositioning of cartilage, or the incorrect migration of neural crest cells that contribute to craniofacial development. Our analysis of the expression of genes that mark migrating neural crest, *sox10* and *crestin*, showed that these genes are essentially expressed normally in *esco2* morphants (Fig. S3A–D). However, the accumulation of pigment cells (Fig. 2F) in *esco2* morphants together with truncated distribution of *crestin* at 16 somites (Fig. S3A, C) suggests that while neural crest cells are specified, they may not be migrating to form the craniofacial cartilage. The cranial neural crest marker *dlx2a* was expressed normally at early stages (Fig. S3E, H), but altered and reduced expression in the pharyngeal arches and forebrain at 24 hpf (Fig. S3F, G, I, J) indicates that *dlx2*-expressing cells may be inefficiently located to these tissues.

**Figure 3:**
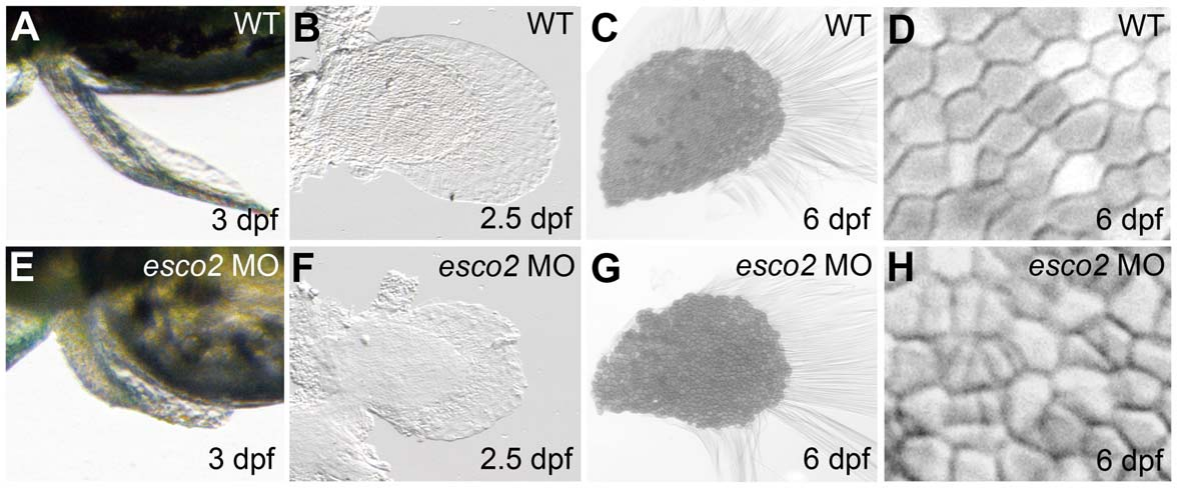
Pectoral fin development is impaired in *esco2* morphants. **A**, **B**, **E**, **F**, The pectoral fin was shorter and abnormally shaped in *esco2* morphants. Fins of wild type (A) and morphant (E) live embryos at 3 dpf (dorsal view). Flat-mounted pectoral fins at 2.5 dpf were smaller and less structured in *esco2* morphants (F) compared with wild type (B). **C**, **G**, **D**, **H**, Alcian Blue staining revealed catch-up growth in pectoral fins at 6 dpf. The fins of *esco2* morphants (G) were comparable to wild type (C) in size and overall structure. Closer views of cell structure in pectoral fins at 6 dpf showed that cells in *esco2* morphant embryos (H) were smaller and more condensed compared to evenly shaped and similar sized cells in wild type (D), perhaps indicating rapid cell divisions in the morphants.

A defining characteristic of RBS is tetraphocomelia [26], therefore we were interested to determine the effects of Esco2 depletion on pectoral fin growth. In 72 hpf *esco2* morphants, the pectoral fin was abnormally shaped and severely reduced in length when compared with wild type (Fig. 3A, E; B, F). Fin reduction in *esco2* morphants was enhanced when the function of p53 was reduced (data not shown). Interestingly, in *esco2* hypomorphants, pectoral fin growth was retarded at first, until around 3 dpf, when presumably, the *esco2* MO becomes too diluted to retain its effect. At this stage, the fins appear to display catch-up growth (Fig. 3C, G). Strikingly, by 6 dpf, cells appeared to be dividing more actively in the developing fins of *esco2* hypomorphants than in wild type embryos (Fig. 3D, H). Our interpretation is that the affected structures appear to compensate for an early paucity of cells by accelerating proliferation once Esco2 levels start to recover. The data suggest that fin stunting is not due to a patterning defect caused by loss of Esco2, but rather by inadequate cell numbers.

### Esco2 morphants exhibit a cell cycle block in G2/M phase

**Figure 4:**
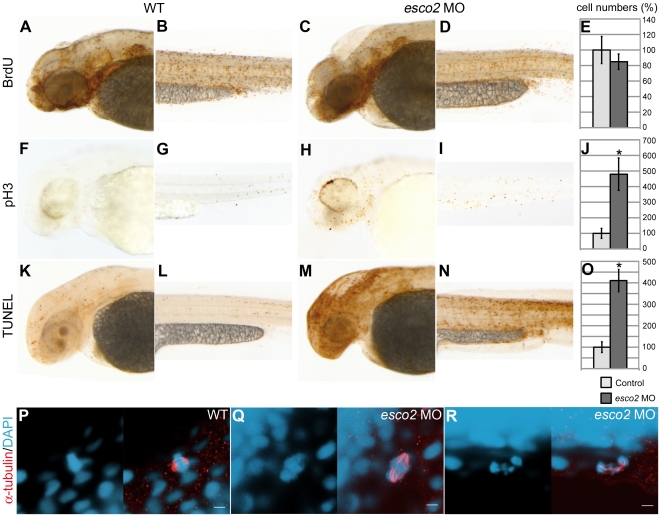
Cell cycle is blocked at mitosis in *esco2* morphants. **A–E**, Cells in S phase stained with BrdU were similar in numbers in wild type (A, B) and *esco2* morphant (C, D). Statistical analyses did not show a significant difference in cell numbers in the tails of embryos at 2 dpf (E). **F–J**, pH3 staining of mitotic cells revealed increased cell numbers in *esco2* morphant (H, I) compared to wild type (F, G). The difference in cell numbers is statistically significant (J). **K–O**, Apoptotic cells labeled with TUNEL were severely increased in *esco2* morphants (M, N, compared to wild type in K, L). The difference in cell numbers is statistically significant (O). Lateral views of 2 dpf larval heads (A, F, K, C, H, M) and regions in tail above the yolk sac extension (B, G, L, D, I, N), anterior to the left. An asterisk in the graphs (J, O) indicates a statistically significant difference in cell numbers of *esco2* morphants compared with wild type embryos (p-value<0.05). **P–R**, Confocal images of mitotic cells at 1 dpf stained with anti-α-tubulin (red) and counterstained with DAPI (blue, left panels). Right panels: merged images. In *esco2* morphants the DNA was fragmented (P, Q, left panels) and spindles were often disorganized (R, right panel) compared to wild type (P). Scale bar: 5 µm.

Loss of craniofacial cartilage and fin stunting in *esco2* morphants could be due to a lack of cell proliferation or cell death in these tissues. To determine the effects of Esco2 depletion on the cell cycle, we labeled S phases with BrdU, M phases with anti-phosphohistone H3 and apoptotic cells with TUNEL. Surprisingly, we found that the number of cells in S phase in *esco2* morphants was roughly equivalent to wild type, indicating that entry into S phase was not compromised by Esco2 depletion (Fig. 4A–E). In contrast, the number of cells in mitosis was significantly increased in *esco2* morphants compared with wild type (Fig. 4F–J). Confocal microscopy revealed that most mitotic cells in *esco2* morphants had distorted spindles, and disorganized chromosomes that were scattered through the cell (Fig. 4P–R). These data indicate that in *esco2* morphants, cells become blocked at the onset of mitosis, which is consistent with the role identified for Esco2 in establishing chromosome cohesion. TUNEL assays indicate high levels of apoptosis in *esco2* morphants (Fig. 4K–O). Apoptotic cells were particularly prevalent throughout the brain, peripheral nervous system and the eyes of *esco2* morphants. The data are consistent with the idea that S phase entry occurs normally in *esco2* morphants, but subsequently, cells are unable to proceed through mitosis and die by apoptosis, which probably reflects the activation of G2 and/or spindle assembly checkpoints in cells of these embryos.

### Apoptosis in *esco2* morphants is associated with caspase activation, but is independent of p53

**Figure 5:**
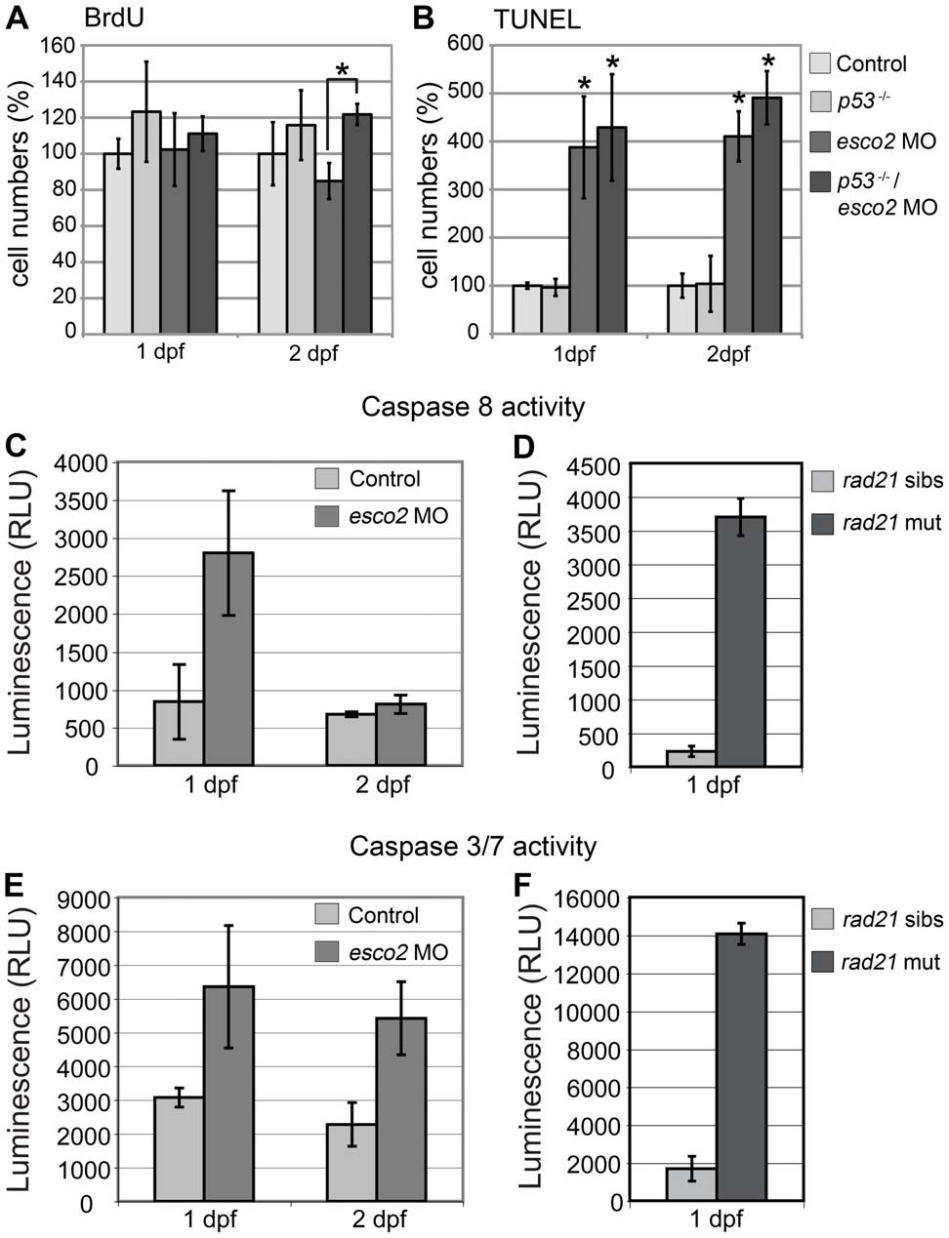
Caspase activity is increased in *esco2* morphants and *rad21* mutants. **A**, Numbers of S phase cells detected by BrdU labeling were similar in *esco2* morphants and wild type at 1 and 2 dpf. Additional depletion of p53 significantly increased S phase entry in *esco2* morphants at 2 dpf (asterisk). **B**, Apoptotic cells labeled with TUNEL were significantly increased in *esco2* morphants compared with wild type at 1 and 2 dpf. Additional depletion of p53 did not rescue the observed apoptosis. Asterisks indicate where a significant difference in cell numbers was observed in *esco2* morphants compared with wild type and the *p53* mutant. **C**, Caspase 8 activity in *esco2* morphants was increased at 1 dpf, but not at 2 dpf compared to wild type control. **D**, Caspase 8 activity was severely increased in *rad21* mutants (*rad21* mut) at 1 dpf compared to siblings (*rad21* sibs). **E**, Caspase 3/7 activity was increased in *esco2* morphants at 1 dpf and 2 dpf. **F**, Caspase 3/7 activity was severely increased in *rad21* mutants at 1 dpf.

To determine if apoptosis in *esco2* morphants is p53-dependent, we injected the *esco2* MO into embryos homozygous for *p53* ^M214K^, a genetic background that leads to compromized p53 function. In the *p53* ^M214K^ line, activation of p53 response genes is absent, therefore p53-dependent apoptosis should not be induced [51], [52]. In this p53-deficient background, cells in S phase were similar between *esco2*-depleted and wild type embryos at 24 hpf (Figs. 5A and S4A–D). However, there was a slight increase of S phases in *esco2* morphants in the p53-deficient background at day 2 (Figs. 5B and S4E–H). This may indicate that intact p53 retards re-entry of damaged *esco2-*depleted cells into the cell cycle. Apoptosis in the *p53* mutant background was just as prevalent as that observed on a wild type background (Figs. 5B and S4I–N). Thus, apoptosis in *esco2* morphants appears to be independent of p53.

One p53-independent pathway for apoptosis is activation of DEATH receptors followed by signaling through caspase 8. Microarray analysis (see below) showed that *casp8* mRNA levels were dramatically increased in *esco2* morphants. To find out if this apoptotic pathway is responsible for the cell death observed in *esco2* morphants, we analyzed morphant embryos for the activity of specific caspases. We found a 2–3 fold enhancement in the activities of caspase 8 (Fig. 5C) and caspases 3 and 7 (Fig. 5E) in 24 hpf *esco2* morphants. By comparison, the activity of these caspases was raised 7–10 fold in 24 hpf *rad21* mutants (Fig. 5E, F). *rad21* mutants are more severely affected than *esco2* morphants at this stage, which could account for this difference. At 2 dpf, only caspases 3/7 remained elevated in *esco2* morphants (Fig. 5E compared with C). The raised caspase activities indicate that caspase-dependent apoptotic programs are indeed activated in *esco2* morphants. The drop in caspase 8 activity by day 2 could indicate that initiation of apoptosis occurs within the first day of Esco2-depleted development.

Together, the data suggest that a paucity of cells in Esco2-depleted embryos is due to increased cell death following a mitotic blockade and activation of DEATH receptor signaling. Because we observed pectoral fin recovery in *esco2* hypomorphants, it is possible that cell death alone could cause the craniofacial and pectoral fin phenotypes. However, it is also possible that these developmental phenotypes are due to the misregulation of developmental genes downstream of *esco2*. Since the related cohesinopathy CdLS is characterized by changes in developmental gene expression, and Esco2 is important for cohesin function, we went on to investigate this possibility.

### Gene regulation downstream of Esco2 has minimal overlap with cohesin-dependent transcription

Several groups have now shown that cohesin has an important role in the regulation of developmental genes [53], [54], [55]. We had previously identified genetic pathways regulated by cohesin during early zebrafish through microarray analysis of a null mutation in the zebrafish *rad21* gene (*rad21* ^nz171^) [37]. This analysis revealed that, in addition to genes involved in the cell cycle, genes involved in transcription and development were regulated downstream of Rad21 [37].

To determine if genes that have altered expression as a result of Esco2 depletion are similar to those regulated downstream of Rad21, we collected RNA from 24 hpf and 48 hpf *esco2* morphants and control embryos, and prepared probes that were hybridized to Affymetrix microarrays. Following normalisation and statistical analysis using linear models (LIMMA; false discovery rate of 5% and |fold change|≥2), differential expression of several transcripts between morphant and wild type embryos was observed, particularly at 24 hpf. Gene Ontology analysis was performed using the GATHER web tool with the 24 hpf differentially abundant transcript lists. This analysis indicated that at 24 hpf, the RNAs differentially expressed in *esco2* morphants were enriched for RNAs involved in cell proliferation and apoptosis: (GO:008283, *ccng1*, *ccng2*, *cdc6*, *cdca7*, *ctbp1*, *cyr61*, *dut*, *erbb2*, *erbb4*, *fgb*, *fgg*, *fhit*, *fhit1*, *fth1*, *irf1*, *mcm2*, *mcm3*, *mdm2*, *mdm4*, *nck1*, *plk3*, *prdx1*, *rps27*, *sesn3*, *tp53*, *tsga2*). Consistent with this cell cycle profile, differentially expressed genes were also enriched for targets of the cell cycle associated transcription factor complex E2F-1:DP-1. However, most of the RNAs that were differentially expressed in the esco2 morphants did not fall into any particular functional category. At 48 hpf, no significant functional pathways emerged from differentially regulated transcripts in *esco2* morphants compared with controls. The data suggest that the transcriptional response to Esco2 depletion happens early, and involves primarily cell cycle regulators along with other RNAs encoding proteins of mixed function.

When the *esco2* array data were compared with our previous analysis of *rad21* ^nz171^ mutants [37], very few of the proliferation- and transcription-associated RNAs affected by Rad21 inactivation were also affected by Esco2-depletion, as indicated by the difference in numbers of colored nodules in the graphs in Figure S5. Taken together the data indicate that, unlike cohesin, Esco2 is probably not directly involved in the transcriptional regulation of developmental and cell cycle genes, at least, not by the same mechanism.

### Regulation of *myca* and *runx* genes by cohesin is not Esco2-dependent

**Figure 6:**
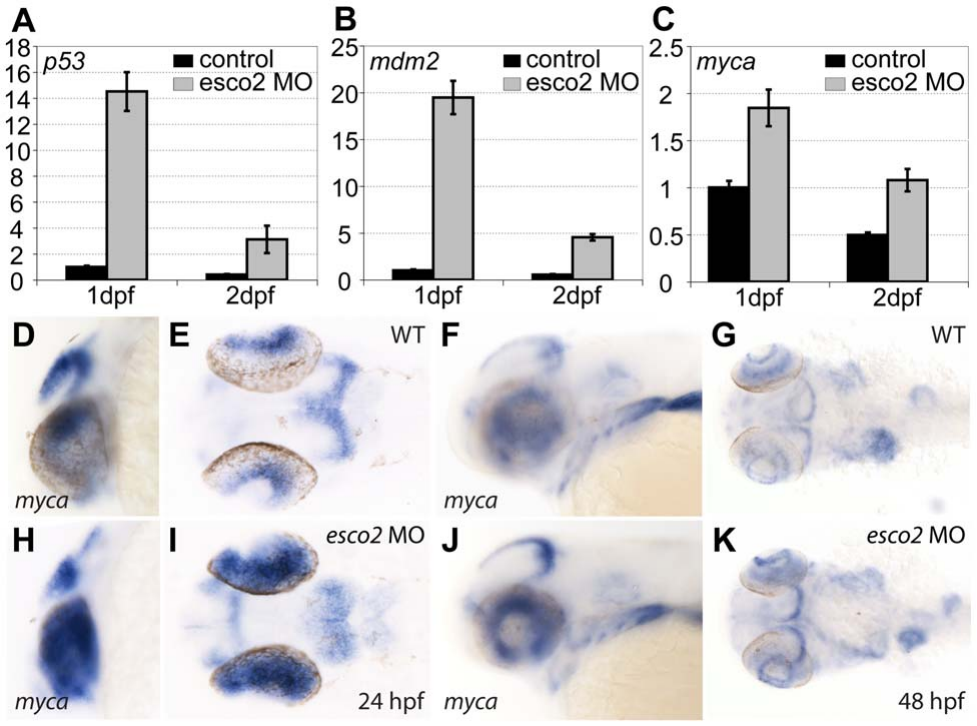
Cell cycle control genes are severely affected in *esco2* morphants. **A–C**, qPCR at 1 and 2 dpf showing significantly increased mRNA abundance in *esco2* morphants of *p53* (A) and *mdm2* (B), and increased mRNA levels of *myca* (C). **D–K**, *In situ* hybridization with a *myca* probe at 24 hpf and 48 hpf. Note the strong expression in the eyes of *esco2* morphants (H) compared with wild type (D) and the abnormal expression in the brain (I compared with wild type in E) at 24 hpf. At 48 hpf *myca* expression seemed to be slightly increased in the brain of *esco2* morphants (J, K compared with wild type in F, G) but was expressed in a normal pattern. D, H, F, J, lateral views, E, I, G, K, dorsal views. Anterior is to the left.

We previously demonstrated that a network of genes related to *myca* (zebrafish *c-Myc*), *mdm2* and *p53* is regulated downstream of Rad21, and that cohesin directly binds the transcription start sites of *myca*, *mdm2*, and *p53* [37]. To test whether these genes are similarly regulated downstream of Esco2, we used quantitative PCR to evaluate their expression in *esco2* morphants at 24 and 48 hpf. Consistent with the microarray results, *p53* and *mdm2* were dramatically upregulated in *esco2* morphants, particularly at 24 hpf (Fig. 6A, B). Surprisingly *myca*, which is strongly downregulated in cohesin-depleted embryos [37], was upregulated up to 2-fold in *esco2* morphants (Fig. 6C, compared to a 1.4-fold increase of *myca* mRNA in the microarray Fig. S5B). This unexpected finding was confirmed by *in situ* hybridization analysis of *myca* expression in whole mount zebrafish embryos. Compared with wild type, a much higher abundance of *myca* transcript was detected in the eyes and brain of 24 and 48 hpf *esco2* morphants (Fig. 6D–K). The reason for *myca* upregulation is not known, but it may be part of a mechanism to compensate for excess cell death in *esco2* morphants (Figs. 4, 5).

The developmental transcription factors *runx1* and *runx3* are regulated by cohesin in a tissue-specific manner [36], so we sought to determine whether these genes are similarly regulated in *esco2* morphants. *In situ* hybridization revealed no change in expression of these genes in *esco2* morphants at the developmental stages in which they are regulated by cohesin (Fig. S6, and data not shown). Therefore we conclude that cohesin-dependent regulation of *myca*, *runx1* and *runx3* does not depend on Esco2 activity.

Together, the results suggest that while there is good evidence that cohesin's regulation of developmental genes could contribute to CdLS, there appears to be no equivalent role for gene regulation by ESCO2 in RBS. If ESCO2 does directly contribute to transcription, it is likely to do so by a distinct mechanism.

## Discussion

While several groups have used animal models to understand the biology of CdLS [34], [35], [56], [57], to date there have been no animal models investigating the pathological basis of RBS. Our study in zebrafish represents the first such example of an animal model. In this study, we have used zebrafish to show that the molecular etiology of RBS is distinct from CdLS.

We found that Esco2 depleted zebrafish embryos exhibit similar phenotypes to humans with RBS [25], [26], [58], [59], [60]. Esco2-depleted fish have craniofacial abnormalities characterized by missing cartilaginous elements (Fig. 2). They have truncated pectoral fins (Fig. 3), much like the phocomelia observed in RBS patients. Cells in Esco2 morphants exhibited mitotic defects characterized by disorganized chromosomes spread throughout the cell (Fig. 4). This observation is consistent with precocious sister chromatid separation observed in cells from RBS patients [60].

Cell death was the most striking phenotype observed in Esco2 morphants (Figs. 4, S4). In the first day of development, Esco2-depleted embryos upregulated genes required for apoptosis, activated cell death pathways and exhibited high numbers of apoptotic cells through the brain and peripheral nervous system (Figs. 4K–O, 5B, C, E; 6A, B; S4). This phenotype was not unexpected, since lack of chromosome cohesion would be certain to activate the spindle assembly checkpoint.

Our results raise the possibility that cell death, rather than defects in patterning, cause dysmorphology upon Esco2 depletion. Pectoral fin growth recovers via a spurt of “catch-up” proliferation in embryos treated with a low dose of *esco2* MO (Fig. 3). This finding strongly suggests that fin stunting in *esco2* morphants is not caused by a patterning defect, and that the underlying limb developmental pathways are unaffected. Ossification is normal in *esco2* morphants (Fig. 2Q, R), further indicating that the developmental pathways giving rise to bone are intact in these embryos.

The lack of jaw elements in *esco2* morphants could be due to cell death in that region, or defects in cell migration from neural crest to form the cartilaginous structures in the jaw. Cells of neural crest origin migrate to form part of the craniofacial skeleton [61]. Markers of neural crest show that these cells are specified normally in *esco2* morphants (Fig. S3). A truncated pattern of *crestin* expression (Fig. S3A, C) and accumulation of patches of pigment in *esco2* morphants (Fig. 2F) suggests that although neural crest contributors to pharyngeal arch development are specified, there may be a problem with their migration to form elements of the craniofacial skeleton (Fig. 2I–P). Alternatively, precursor cells may be lost due to apoptosis, and thereby lose the opportunity to migrate. Interestingly, cell death, cell migration defects and jaw structure loss were found upon mutation of another chromatin modifying factor, *phf8* [62]. Perhaps Esco2 is involved in histone modification pathways similar to Phf8, or alternatively, both phenotypes may reflect cell death in the developing embryo.

It was previously shown that developmental defects arise with abnormalities in cell proliferation, or an increase in cell death, as the sole cause. For example, an X-irradiation driven chick model of phocomelia showed that loss of proximal structures is caused by a time-dependent loss of skeletal progenitors, and not by a patterning defect [63]. Phocomelia is a consistent feature of RBS, and our zebrafish data support the idea that this feature need not be due to patterning defects.

It has often been argued that cohesinopathies arise from changes in developmental gene expression that result from cohesin's role in gene regulation. Previous work has shown that several genes are up- and down- regulated in response to cohesin loss [35], [37], [38], [40], [64]. Changes in developmental gene expression downstream of cohesin are thought to contribute to the pathology of CdLS [50]. A similar function for ESCO2 in causing RBS has not yet been identified. Our microarray data demonstrated that few specific developmental pathways are co-ordinately regulated downstream of Esco2, and that the regulated RNAs in *esco2* morphants are primarily involved in apoptosis and the cell cycle. These results are in sharp contrast with those obtained previously in *rad21* ^nz171^ mutants, where dense networks of cell cycle-associated RNAs emerged [37] (Fig. S5). The transcriptional regulators *runx1*, *runx3* and *myca* genes are all positively regulated by cohesin in zebrafish [36], [37], but are not regulated by Esco2 in the same direction (Figs. 6C–K, S6 and data not shown). In fact, *myca* is upregulated upon Esco2 depletion (Fig. 6C–K), possibly to compensate for reduced proliferation. Importantly, while the loss of acetyltransferase activity of Esco2 can cause RBS [24], it is not required for the regulation of cohesin-dependent genes. Thus, the cohesin acetylating activity of Esco2 can be separated from any other role it might have in regulating transcription. If Esco2 is responsible for direct regulation of gene expression, it probably does so independently of cohesin.

Although we found that few of the RNAs were regulated by cohesin disruption (outside of those involved in the cell cycle and apoptosis) were also regulated by Esco2 disruption, there were notable exceptions. One of these was *ascl1*, a Notch-responsive gene that promotes neuronal differentiation [65]. Interestingly, Ascl1/Mash1 was found to be one of only three factors needed to convert fibroblasts into neuronal progenitors [66]. We found that, in addition to being downregulated upon Esco2 depletion, *ascl1* expression is exquisitely sensitive to the gene dose of *rad21* [37] and it is also downregulated on depletion of zebrafish Nipbl (M.M. and J.A.H., unpublished data). This raises the possibility that there are additional, as yet unknown pathways downstream of cohesion proteins linking cell proliferation with differentiation.

In summary, our results indicate that the molecular pathology underlying RBS is unlikely to involve the regulation of developmental genes, at least not by similar mechanisms to those proposed for CdLS. This draws into question the use of “cohesinopathies” to describe a class of syndromes with a similar genetic etiology, but distinct developmental pathologies.

## Materials and Methods

### Zebrafish lines

Zebrafish were maintained as described previously [67]. All zebrafish research was approved by the University of Otago Animal Ethics Committee (approval #07/54).

### Microinjection

Antisense morpholino oligonucleotides were obtained from GeneTools LLC. For microinjection, 2 nl of morpholino solution diluted in Danieau's buffer was injected into the yolk of wild type embryos at the 1 to 2-cell stage. Morpholino sequences and effective amounts were *esco2*_ATG_MO, 5′-CTCTTTCGGGATAACATCTTCAATC-3′ (0.175-0.25 pmol) and *esco2*_splx2_MO, 5′-GTAAACTACACAATGTTACCTCTCG-3′ (0.75-1.25 pmol).

### In situ hybridization and morphological analysis

A 1120 bp long fragment of *esco2* (accession number NM_001003872) was amplified with 5′-AGCAGGGACCTTCTACAGCA-3′ forward and 5′-GGGATCATCTGGAAGAACGA-3′ reverse primers, and cloned into the pGEM-Teasy vector (Promega) for riboprobe synthesis. The plasmid was linearized with *Sac*II (Roche) and transcribed with SP6 RNA polymerase (Roche). *In situ* hybridization was performed as described previously [68]. Cartilage and bone was stained with Alcian Blue and Alizarin Red, respectively, as described previously [69].

### Cell cycle analysis and immunohistochemistry

Bromodeoxyuridine (BrdU) incorporation was performed on whole embryos as described previously [70]. Mouse monoclonal anti-BrdU antibody (Roche) was detected using a Vectastain ABC Kit (Vector Laboratories). Phosphorylated histone H3 was detected as described previously using an anti-Phospho-Histone H3 (Ser10) antibody (Cell Signaling), followed by detection with anti-rabbit-HRP (Sigma) [70]. TUNEL staining was performed using the ApopTag Kit (Chemicon) as described previously [70]. Cell counts were done on images of a defined region of the tail of 3 embryos each. The cell number of the wild type was set to 100%. To statistically compare cell counts between the samples, a two-sided *t*-test was used. Spindle morphology in mitotic cells was analyzed on whole embryos with anti-α-tubulin (Sigma), and anti-rabbit-TRITC (Sigma) antibodies as previously described [70]. DNA was counterstained with DAPI (Invitrogen), embryos mounted in Vectashield (Vector laboratories) and analyzed by confocal microscopy (Zeiss LSM 510).

### Quantitative RT-PCR

Total RNA from pools of 50 embryos was extracted using Trizol, DNAse-treated, and used to synthesize random-primed cDNA (SuperScriptIII, Invitrogen). SYBR Green PCR Master Mix (Applied Biosystems) was used to amplify cDNA, and relative quantities were normalized to *β-actin* and *wnt5a* expression. Samples were analyzed using an Applied Biosystems 7300 Real-Time PCR System. Quantitative PCR primer sequences for *mdm2*, *p53* and *mcya* were reported previously [37].

### Caspase assays

Single cell suspensions were generated in triplicates of 25 embryos at the desired stage by mashing and filtering in PBS/10% NCS through cell strainers (100 µm, BD Falcon). Cells were resuspended in PBS/10% NCS and suspensions used in Caspase-Glo 8 and Caspase-Glo 3/7 Assays (Promega) according to the manufacturer's instructions. To reduce non-specific background activity in the Caspase 8 assay, the provided inhibitor MG-132 (Promega) was used according to the manufacturer's instructions. Luminescence was measured after 75 minutes on a Synergy 2 multi-detection microplate reader (BioTek Instruments, Inc.). Protein concentration was assessed using a BCA Protein Assay Kit and a BSA standard (Pierce) according to the manufacturer's instructions and was similar in all samples (not shown).

### Microarray and analysis

Total RNA was extracted from 4 replicates (50 embryos each) of 24 hpf and 48 hpf *esco2*_ATG morphants and control embryos (injected with buffer only) using Trizol (Invitrogen) and purified using Qiagen RNeasy columns. RNA integrity was measured using the Agilent Bioanalyzer 2100 (RIN ≥9.5 for all samples). Hybridization to GeneChip Zebrafish Genome Arrays (Affymetrix) and data acquisition were performed at The University of Otago Genomics Facility. All microarray data is MIAME compliant and the raw data has been deposited in GEO, accession number GSE27569. Analysis was performed using the statistical framework ‘R’ (http://cran.r-project.org/). All microarrays passed quality control using the AffyQCreport package. Data were normalized using the RMA method and differential RNA abundance identified using LIMMA. The thresholds for the generation of lists of differentially expressed RNAs were chosen after observation of heatmaps, and the false discovery rate was controlled at 5% using the Benjamini and Hochberg method, with the additional criteria that |mean linear fold change|≥2. Gene sets were tested for enrichment of particular functional categories using the GATHER web tool (http://gather.genome.duke.edu/) with Bayes Factor cutoff ≥5 and p≤0.05, as well as using the Ingenuity Pathways Analysis software (http://www.ingenuity.com/).

## Supporting Information

Figure S1 **Multiple sequence alignment of Esco2 sequences.** Protein sequences of human, mouse and zebrafish were aligned using the ClustalW program (www.ebi.ac.uk/clustalw/; Chenna et al., 2003, PubMedID: 12824352). Accession numbers for sequences used are *Homo sapiens* Q56NI9.1, *Mus musculus* Q8CIB9.3, and *Danio rerio* Q5SPR8.1.
(TIF)

Figure S2 **Evolutionary conservation and knock down of zebrafish** ***esco2.*** **A**, Cladograms of Esco1 and Esco2 proteins. Cladograms were constructed using Geneious Pro 4.6.2 software. Accession numbers for protein sequences are *S. cerevisiae* CAY79478, *D. melanogaster* AAF50579, for Esco2: *D. rerio* Q5SPR8, *S. salar* ACI33242, *X. laevis* BAF91194, *G. gallus* XP_420012, *M. musculus* Q8CIB9, *R. norvegicus* XP_002725161, *H. sapiens* Q56NI9, *P. troglodytes* XP_001164762, *B. taurus* NP_001094652, *A. melanoleuca* XP_002914478, and for Esco1: *X. laevis* ADP44706, *M. musculus* Q69Z69, *R. norvegicus* AAI66441, *M. mulatta* XP_001091733, *P. troglodytes* XP_523883, *H. sapiens* Q5FWF5, *A. melanoleuca* XP_002928389, *E. caballus* XP_001491308, *S. scrofa* XP_003127900, *B. taurus* DAA15944. **B**, Conserved synteny of the zebrafish *esco2* gene when compared with human and mouse. Databases: Ensembl Danio rerio version 59.8 (Zv8), Ensembl Homo sapiens version 59.37d (GRCh37), and Ensembl Mus musculus version 59.37l (NCBIM37). **C**, The esco2_splx2_MO inhibits splicing of the second intron. Primers spanning intron 2 were used to detect splice blocking. cDNA, complementary DNA; control, no reverse transcriptase; WT, cDNA from uninjected embryos; MO, cDNA from esco2_splx2_MO-injected embryos; gDNA, genomic DNA where amplification product size is identical to the unspliced transcript.
(TIF)

Figure S3 **Some neural crest and developmental markers are expressed in a slightly abnormal pattern in** ***esco2*** **morphants.** **A**, **C**, *Crestin* expression at the 16 somite stage. *Crestin* expressing cells at the anterior side were more condensed in *esco2* morphants (C, arrows) than wild type (A). **B**, **D**, *Sox10* expression was normal in *esco2* morphants at the 15 somite stage (D, compared to wild type in B). **E–J**, *Dlx2a* expression at 16 somites was normal in *esco2* morphants (H, compared to wild type in E). At 24 hpf expression of *dlx2a* was slightly reduced in the branchial arches and the forebrain of *esco2* morphants (I, compared to F). The close-ups on the forebrain show an abnormal *dlx2a* distribution in *esco2* morphants (J, compared to wild type in G).
(TIF)

Figure S4 **Apoptosis in** ***esco2*** **morphants is independent of** ***p53*** **.** **A–H**, BrdU-labeled proliferating cells at 24 hpf (A–D) and 48 hpf (E–H). Proliferation was normal in *esco2* morphants (B, F, compare to wild type in A, E). Additional depletion of *p53* in the *p53* ^M214K^ mutant line (p53^−/−^, C, G) led to increased proliferation at 48 hpf (H), but not 24 hpf (D). **I–N**, TUNEL staining of apoptotic cells at 24 hpf (I, J) and 48 hpf (K–N). Apoptosis was severely increased in *esco2* morphants (J, L, compared to wild type in I, K), and independent of additional depletion of *p53* (M, N).
(TIF)

Figure S5 **Ingenuity pathway analysis of** ***rad21*** **mutants and** ***esco2*** **morphants.** Ingenuity pathway analysis (http://www.ingenuity.com/products/pathways_analysis.html) of *rad21* mutants (A) and *esco2* morphants (B) at 1 dpf. Only a subset of Rad21-regulated genes (e.g. those in the *mcm* cluster at the bottom right) were also regulated by Esco2 depletion. The cut-off used for the Rad21 experiment (A) is |unlogged fold change| ≥1.5 and q(FDR-adjusted p) ≤0.01. The same graph is shown for the Esco2 experiment, but colored according the degree of RNA regulation in the *esco2* array (B). Fold changes are overlaid in both graphs. Red, up-regulated in *rad21* mutants or *esco2* morphants, respectively, relative to controls; green, down-regulated in *rad21* mutants or *esco2* morphants, respectively, relative to controls.
(TIF)

Figure S6 ***Runx1*** **expression is normal in** ***esco2*** **morphants.** *Runx1* expression at 12–13 somites was normal in *esco2* morphants (B, compared to wild type in A) compared to missing expression in hematopoietic progenitor cells in *rad21* ^nz171^ mutants (C, [36]).
(TIF)
