# Supplementary figures and images for: A Zebrafish Model of Roberts Syndrome Reveals That Esco2 Depletion Interferes with Development by Disrupting the Cell Cycle
Source: PLoS One. 2011 May 26;6(5):e20051. doi: 10.1371/journal.pone.0020051 (PMC3102698; doi:10.1371/journal.pone.0020051)

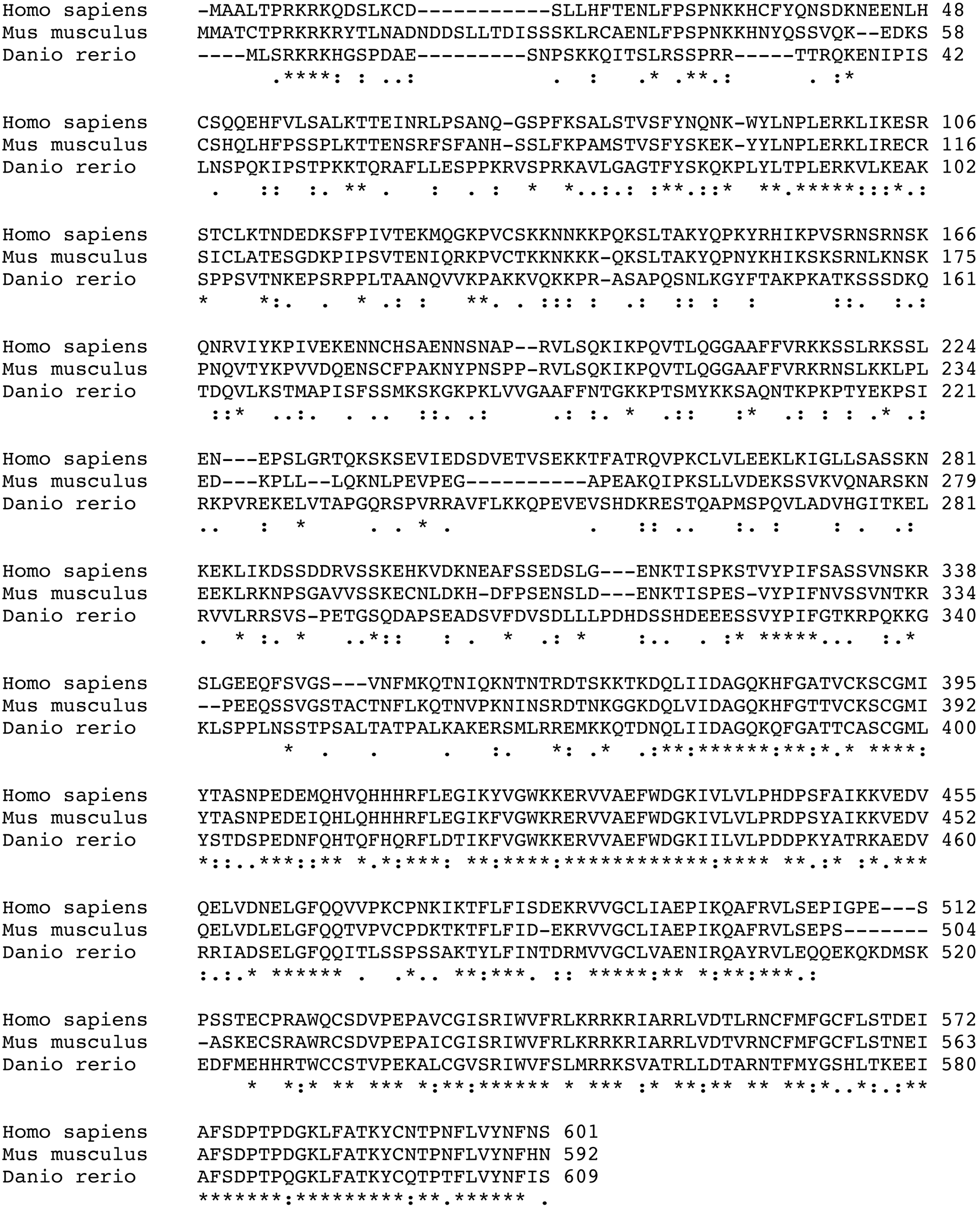

Supplement: Figure S1 — Multiple sequence alignment of Esco2 sequences. Protein sequences of human, mouse and zebrafish were aligned using the ClustalW program (www.ebi.ac.uk/clustalw/; Chenna et al., 2003, PubMedID: 12824352). Accession numbers for sequences used are Homo sapiens Q56NI9.1, Mus musculus Q8CIB9.3, and Danio rerio Q5SPR8.1. (TIF) [file pone.0020051.s001.tif]

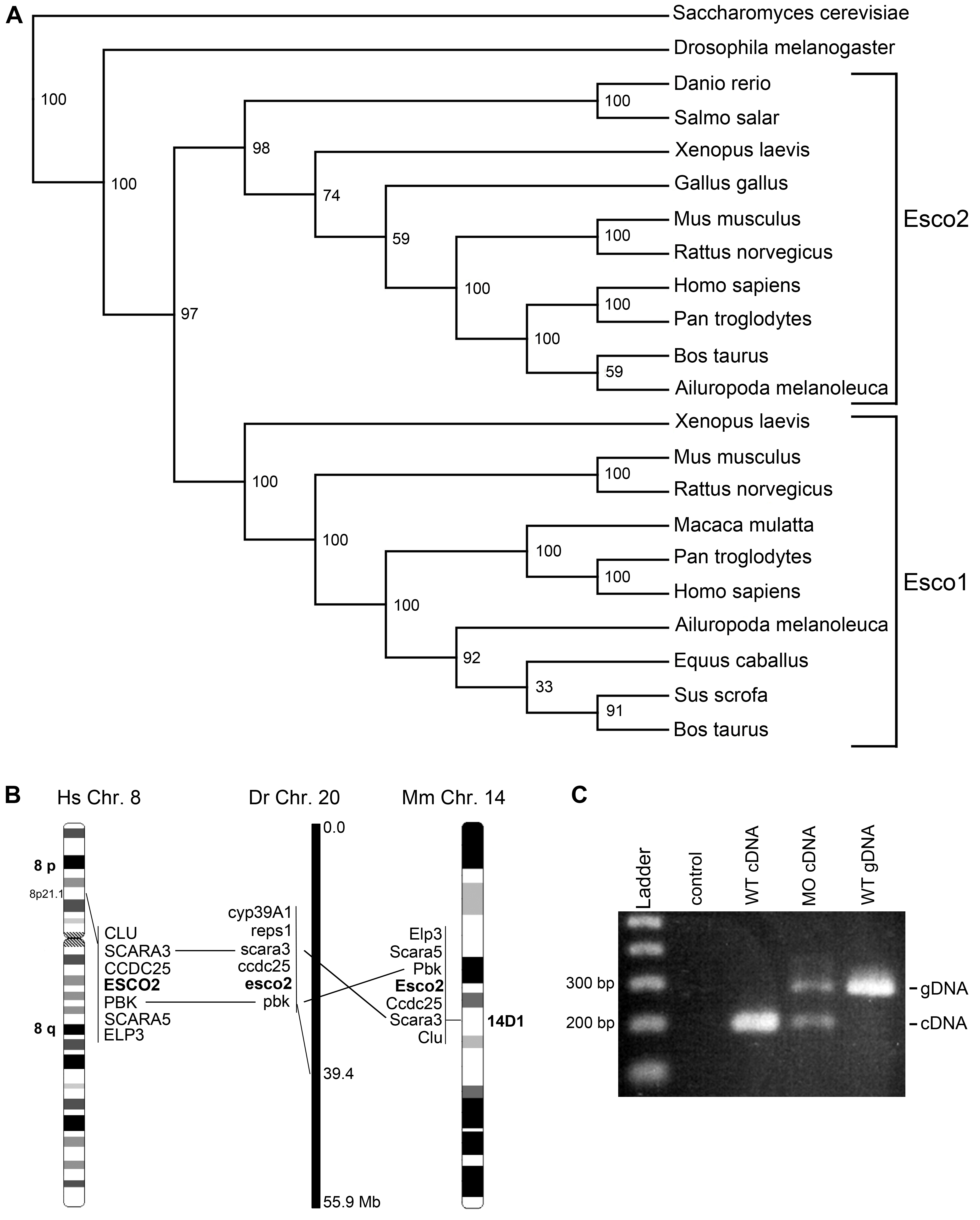

Supplement: Figure S2 — Evolutionary conservation and knock down of zebrafish esco2. A, Cladograms of Esco1 and Esco2 proteins. Cladograms were constructed using Geneious Pro 4.6.2 software. Accession numbers for protein sequences are S. cerevisiae CAY79478, D. melanogaster AAF50579, for Esco2: D. rerio Q5SPR8, S. salar ACI33242, X. laevis BAF91194, G. gallus XP_420012, M. musculus Q8CIB9, R. norvegicus XP_002725161, H. sapiens Q56NI9, P. troglodytes XP_001164762, B. taurus NP_001094652, A. melanoleuca XP_002914478, and for Esco1: X. laevis ADP44706, M. musculus Q69Z69, R. norvegicus AAI66441, M. mulatta XP_001091733, P. troglodytes XP_523883, H. sapiens Q5FWF5, A. melanoleuca XP_002928389, E. caballus XP_001491308, S. scrofa XP_003127900, B. taurus DAA15944. B, Conserved synteny of the zebrafish esco2 gene when compared with human and mouse. Databases: Ensembl Danio rerio version 59.8 (Zv8), Ensembl Homo sapiens version 59.37d (GRCh37), and Ensembl Mus musculus version 59.37l (NCBIM37). C, The esco2_splx2_MO inhibits splicing of the second intron. Primers spanning intron 2 were used to detect splice blocking. cDNA, complementary DNA; control, no reverse transcriptase; WT, cDNA from uninjected embryos; MO, cDNA from esco2_splx2_MO-injected embryos; gDNA, genomic DNA where amplification product size is identical to the unspliced transcript. (TIF) [file pone.0020051.s002.tif]

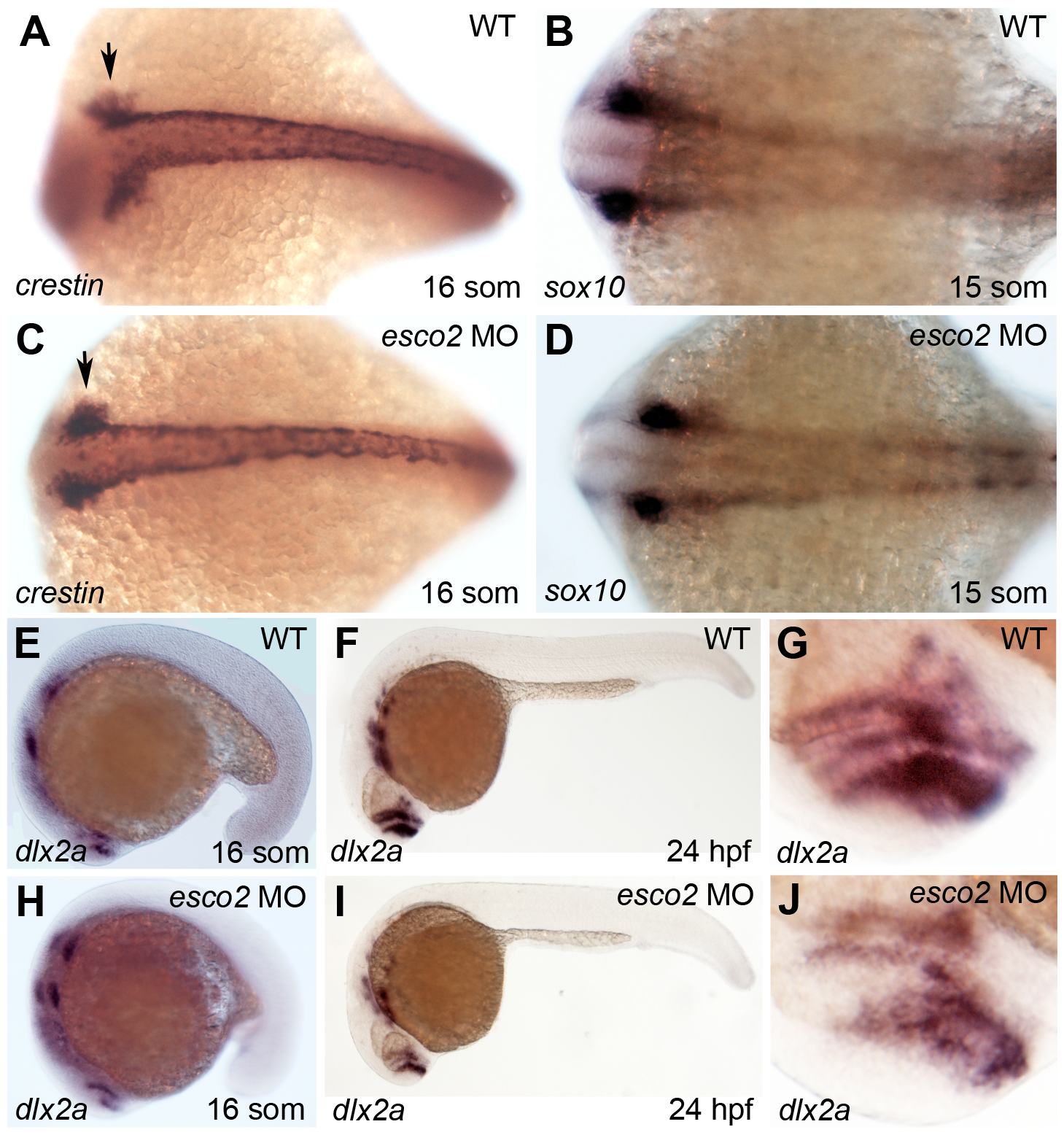

Supplement: Figure S3 — Some neural crest and developmental markers are expressed in a slightly abnormal pattern in esco2 morphants. A, C, Crestin expression at the 16 somite stage. Crestin expressing cells at the anterior side were more condensed in esco2 morphants (C, arrows) than wild type (A). B, D, Sox10 expression was normal in esco2 morphants at the 15 somite stage (D, compared to wild type in B). E–J, Dlx2a expression at 16 somites was normal in esco2 morphants (H, compared to wild type in E). At 24 hpf expression of dlx2a was slightly reduced in the branchial arches and the forebrain of esco2 morphants (I, compared to F). The close-ups on the forebrain show an abnormal dlx2a distribution in esco2 morphants (J, compared to wild type in G). (TIF) [file pone.0020051.s003.tif]

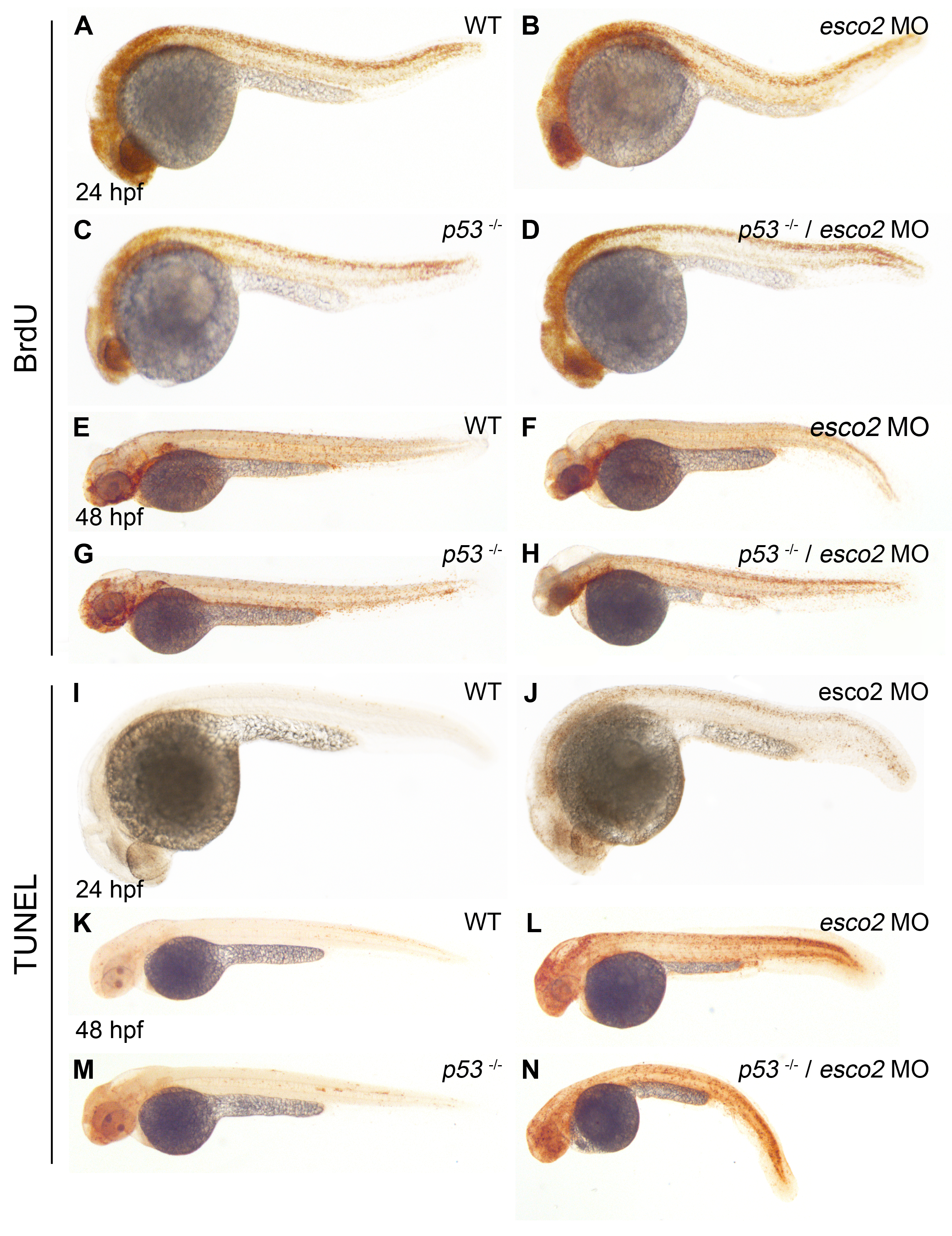

Supplement: Figure S4 — Apoptosis in esco2 morphants is independent of p53 . A–H, BrdU-labeled proliferating cells at 24 hpf (A–D) and 48 hpf (E–H). Proliferation was normal in esco2 morphants (B, F, compare to wild type in A, E). Additional depletion of p53 in the p53 M214K mutant line (p53−/−, C, G) led to increased proliferation at 48 hpf (H), but not 24 hpf (D). I–N, TUNEL staining of apoptotic cells at 24 hpf (I, J) and 48 hpf (K–N). Apoptosis was severely increased in esco2 morphants (J, L, compared to wild type in I, K), and independent of additional depletion of p53 (M, N). (TIF) [file pone.0020051.s004.tif]

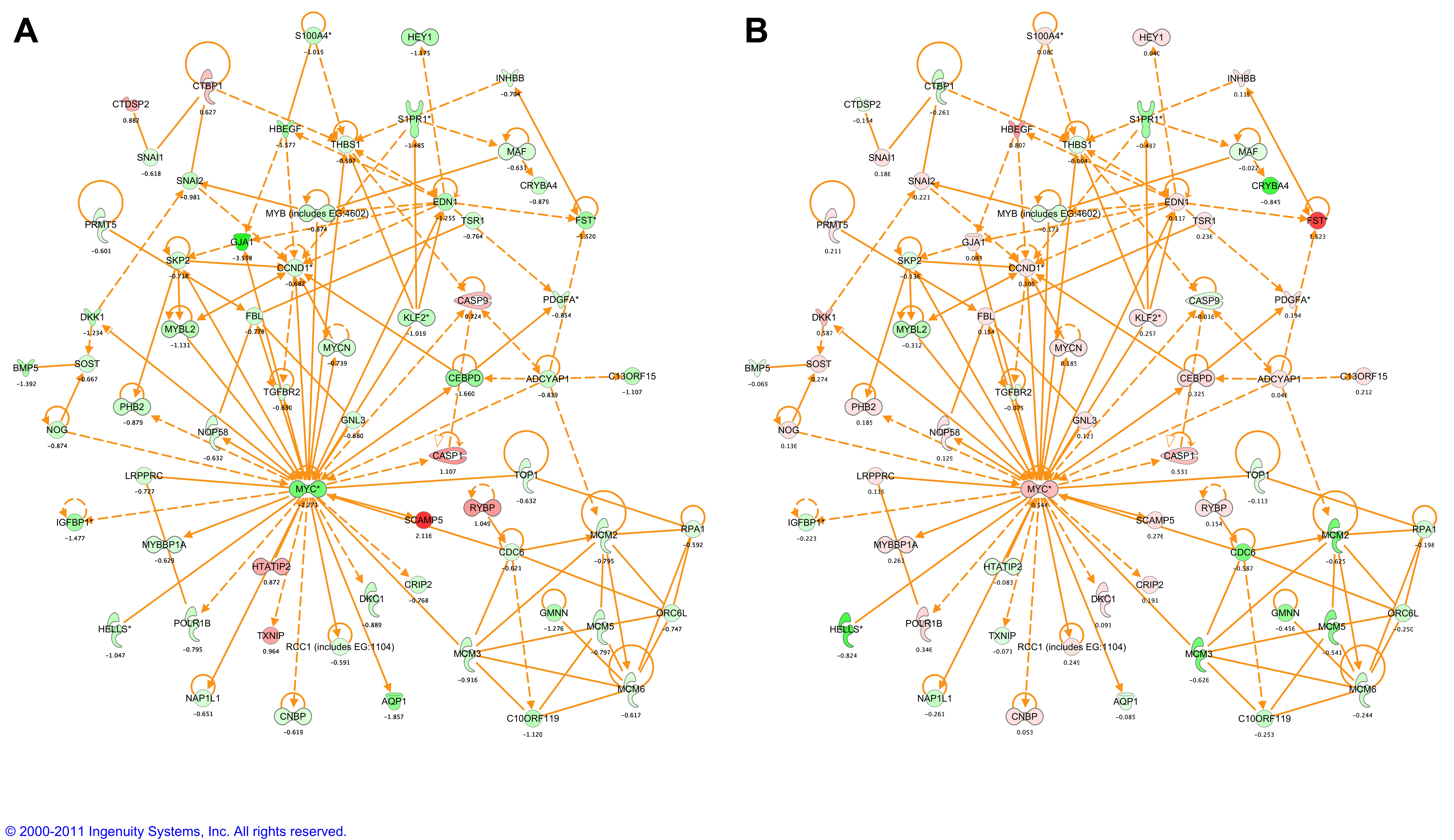

Supplement: Figure S5 — Ingenuity pathway analysis of rad21 mutants and esco2 morphants. Ingenuity pathway analysis (http://www.ingenuity.com/products/pathways_analysis.html) of rad21 mutants (A) and esco2 morphants (B) at 1 dpf. Only a subset of Rad21-regulated genes (e.g. those in the mcm cluster at the bottom right) were also regulated by Esco2 depletion. The cut-off used for the Rad21 experiment (A) is |unlogged fold change| ≥1.5 and q(FDR-adjusted p) ≤0.01. The same graph is shown for the Esco2 experiment, but colored according the degree of RNA regulation in the esco2 array (B). Fold changes are overlaid in both graphs. Red, up-regulated in rad21 mutants or esco2 morphants, respectively, relative to controls; green, down-regulated in rad21 mutants or esco2 morphants, respectively, relative to controls. (TIF) [file pone.0020051.s005.tif]

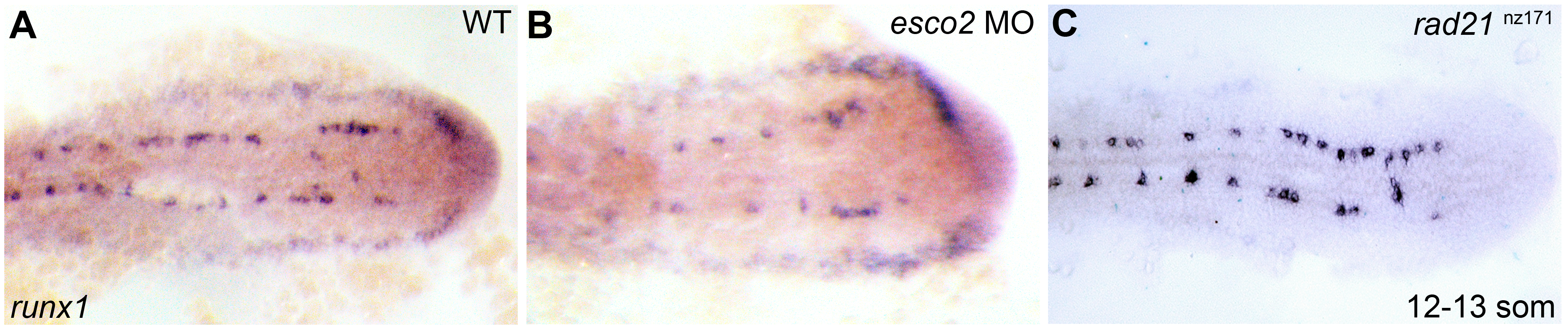

Supplement: Figure S6 — Runx1 expression is normal in esco2 morphants. Runx1 expression at 12–13 somites was normal in esco2 morphants (B, compared to wild type in A) compared to missing expression in hematopoietic progenitor cells in rad21 nz171 mutants (C, [36]). (TIF) [file pone.0020051.s006.tif]
